# Supplementary material for: ROP16 of Toxoplasma gondii Inhibits Innate Immunity by Triggering cGAS-STING Pathway Inactivity through the Polyubiquitination of STING
Source: Cells. 2023 Jul 15;12(14):1862. doi: 10.3390/cells12141862 (PMC10378213; doi:10.3390/cells12141862)
Supplement: Supplementary file 1 [file cells-12-01862-s001.zip › cells-2455989-supplementary.pdf]

Supporting Information

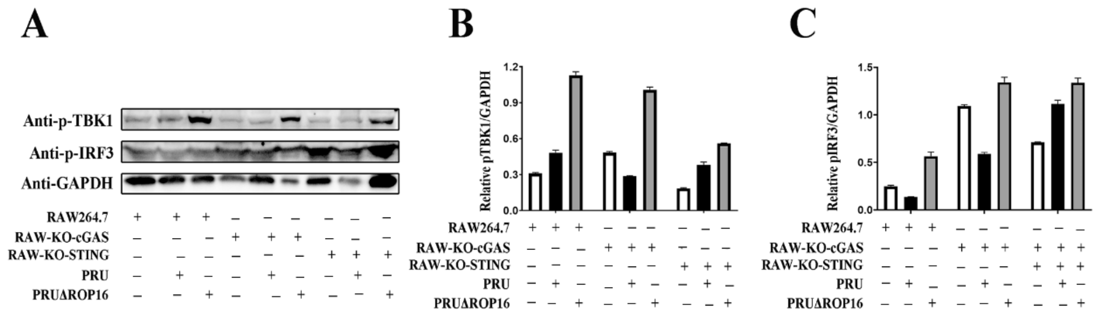

FIGURE S1 | Knockdown of ROP16 increases *T.gondii*-triggered cGAS-STING signaling

RAW264.7, RAW-KO-STING and RAW-KO-cGAS cells were infected with PRU or PRUΔROP16 tachyzoites. 16 hours past infection the infected cells were harvested for Western Blotting assays. (A)Western Blotting assayed the activity of cGAS-STING signaling by detecting the phosphorylated IRF3 and phosphorylated TBK1. (B) Image J densitometry analysis for pTBK1 relative to GAPDH and for pIRF3 relative to GAPDH(C).Data were presented as mean±SEM of three independent experiments.

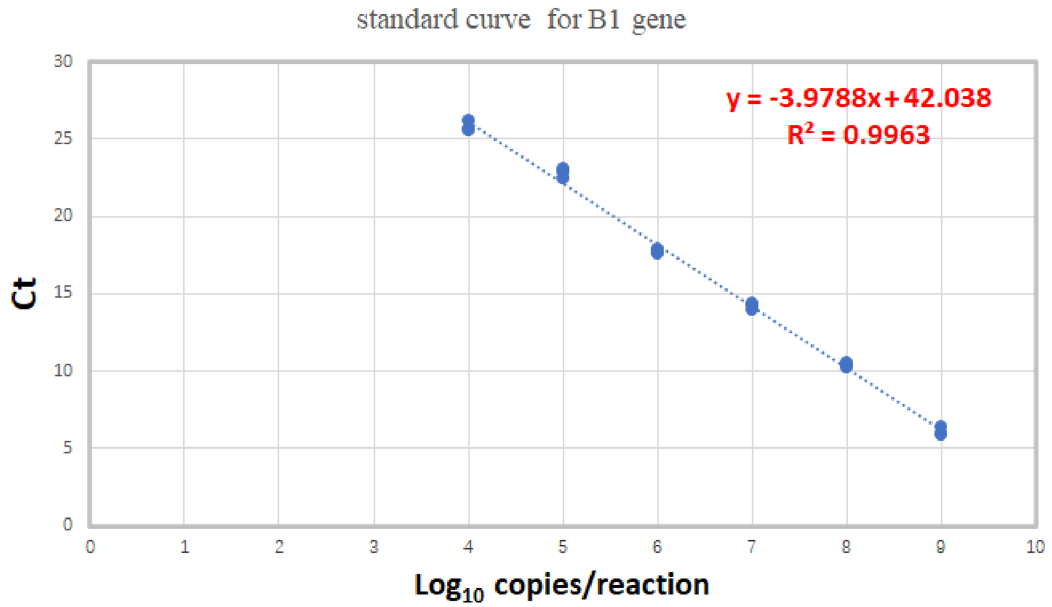

FIGURE S2 | Standard curve generated from qPCR amplification of known numbers of target (threshold cycle [Ct] versus log10 copies/reaction).

Table S1 | primers used in this study

Primers	Sequences from 5' to 3'	Used to
ROP16-F<br>ROP16-R	CCAGAATTCATGAAAGTGACCACGAAAGGGCTTG<br>AATGGGCCCCTACATCCGATGTGAAGAAAG	Amplify coding sequence of ROP16
mcGAS-F<br>mcGAS-R	AATGGTACCATGGAAGATCCGCGTAGAAGGACG<br>CCACTCGAGTCAAAGCTTGTCAAAAATTGGAAAC	Amplify coding sequence of cGAS
mSTING-F<br>mSTING-R	CCAGAATTCTATGCCATACTCCAACCTGCATCCAG<br>TTACTCGAGTCAGATGAGGTCAGTGCGGAGTG	Amplify coding sequence of STING
mTBK1-F<br>mTBK1-R	ATCGGATCCATGCAGAGCACCTCCAACCAT<br>AATGGGCCCCTAAAGACAGTCCACATTGCG	Amplify coding sequence of TBK1
mIRF3-F<br>mIRF3-R	CCAGAATTCTATGGAAACCCCGAAACCGCG<br>CCACTCGAGTCAGATATTTCCAGTGGCCTG	Amplify coding sequence of IRF3
R16-24aaF	CCAGAATTCCATGCGATACATGTCGTTTGAGGAAGC	Amplify coding sequence of truncated ROP16
R16-345aaF	CCAGAATTCCATGCAACCTCCAGGAGCGGTGGAGTC	Amplify coding sequence of truncated ROP16
R16-401aaF	CCAGAATTCCATGTATGCAGCGAAGGTGCCGTACAG	Amplify coding sequence of truncated ROP16
R16-533aaR	AATGGGCCCTCCAAGTTCGTGAAGCTTGGCCAAT	Amplify coding sequence of truncated ROP16
qIFN-β-F<br>qIFN-β-R	CAGCTCCAAGAAAGGACGAAC<br>GGCAGTGTAACTCTTCTGCAT	qPCR analyses the expression of IFN-β
qIL-12b-F<br>qIL-12b-R	TGGTTTGCCATCGTTTTGCTG<br>ACAGGTGAGGTTCACTGTTTCT	qPCR analyses the expression of IL-12b
qIL-6-F<br>qIL-6-R	TAGTCCTTCCTACCCCAATTTCC<br>TTGGTCCTTAGCCACTCCTTC	qPCR analyses the expression of IL-6
qCXCL-10-F<br>qCXCL-10-R	CCAAGTGCTGCCGTCATTTTC<br>GGCTCGCAGGGATGATTTCAA	qPCR analyses the expression of CXCL10
qISG15-F<br>qISG15-R	GGTGTCCGTGACTAACTCCAT<br>TGGAAAGGGTAAGACCGTCCT	qPCR analyses the expression of ISG15
qGAPDH-F<br>qGAPDH-R	AGGTCGGTGTGAACGGATTTG<br>TGTAGACCATGTAGTTGAGGTCA	Amplify housekeeping gene
gRNA-F<br>gRNA-R	GCTCACACTTACAGAGACTGGTTTTAGAGCTAGAA<br>AACTTGACATCCCCATTTAC	Construct gRNA plasmid for localization of ROP16
pUC19-F<br>pUC19-R	GGCGTAATCATGGTCATAGC<br>ACTGGCCGTCGTTTTACAAC	Amplify pUC19 linerized vector
DHFR-F<br>DHFR-R	CAACCCGCGCAGAAGACATC<br>GGACACGCTGAACTTGTGGC	Amplify DHFR-UTR sequence
5'UTR-F<br>5'UTR-R	GTTGTAAAACGACGGCCAGTGCCTGAGACCCATGTT<br>GATGTCTTCTGCGCGGGTTGCTTGCGACAAACAAGA	Amplify homologous template of ROP16
3'UTR-F<br>3'UTR-R	GCCACAAGTTCAGCGTGTCCGGTGTAAGGTTCCCAC<br>GCTATGACCATGATTACGCCGCCATCCAACGCCTTC	Amplify homologous template of ROP16
PCR1-F	TCAGTGTAGAAGGAATCCGC	Identify ROP16 knockout

PCR1-R	ATTTGTGAGGACGACTCACG	strain
PCR2-F<br>PCR2-R	AGGATGAATTCCTACCCAGC<br>CTGTTTCTCGGTCCAAGACA	Identify ROP16 knockout strain
PCR3-F<br>PCR3-R	CGATACATGTCGTTTGAGGAAGCGC<br>TCCAAGTTCGTGAAGCTTGGCCAAT	Identify ROP16 knockout strain
